# Generating and testing the efficacy of reagents for CRISPR/Cas9 homology directed repair-based manipulations in *Tribolium*

**DOI:** 10.1093/jisesa/ieae082

**Published:** 2024-08-20

**Authors:** Hannah C Markley, Kennedy J Helms, Megan Maar, Gabriel E Zentner, Michael J Wade, Andrew C Zelhof

**Affiliations:** Department of Biology, Indiana University, Bloomington, IN, USA; Department of Biology, Indiana University, Bloomington, IN, USA; Department of Biology, Indiana University, Bloomington, IN, USA; Department of Biology, Indiana University, Bloomington, IN, USA; Department of Biology, Indiana University, Bloomington, IN, USA; Department of Biology, Indiana University, Bloomington, IN, USA

**Keywords:** CRISPR/Cas9, Tribolium, homology directed repair, non-homologous end joining, gene drive

## Abstract

CRISPR/Cas9 manipulations are possible in many insects and ever expanding. Nonetheless, success in one species and techniques developed for it are not necessarily applicable to other species. As such, the development and expansion of CRISPR-based (clustered regularly interspaced short palindromic repeats) genome-editing tools and methodologies are dependent upon direct experimentation. One useful technique is Cas9-dependent homologous recombination, which is a critical tool for studying gene function but also for developing pest related applications like gene drive. Here, we report our attempts to induce Cas9 homology directed repair (HDR) and subsequent gene drive in *Tribolium castaneum* (Herbst; Insecta: Coleoptera: Tenebrionidae). Utilizing constructs containing 1 or 2 target gRNAs in combination with Cas9 under 2 different promoters and corresponding homology arms, we found a high incidence of CRISPR/Cas9 induced mutations but no evidence of homologous recombination. Even though the generated constructs provide new resources for CRISPR/Cas9 modification of the *Tribolium* genome, our results suggest that additional modifications and increased sample sizes will be necessary to increase the potential and detection for HDR of the *Tribolium* genome.

## Introduction

CRISPR/Cas9 homology directed repair (HDR) has become an invaluable tool for studies of gene function (Shalem et al. 2015, Wang et al. 2016, Khadempar et al. 2019) and serve as the basis for gene drive systems that have increased the possibility of devising synthetic methodologies, versus utilizing naturally occurring selfish elements, for controlling insect pests and limiting the spread of disease vectors (McFarlane et al. 2018, Asad et al. 2022, Bier 2022). CRISPR based HDR has been demonstrated in several insect species and subsequent gene drives have now been demonstrated in *Drosophila* and mosquitos, both *Anopheles* and *Aedes* (Gantz and Bier 2015, Gantz et al. 2015, Hammond et al. 2016, Kyrou et al. 2018, Li et al. 2020). Success and ability to drive through a population is dependent on numerous factors (e.g., diploid versus diploid/haploid genomes (Scott et al. 2018), nature (e.g., sterility, viability, maternal effect (Backus and Delborne 2019)) and timing of drive-gene expression (Burt and Crisanti 2018), mating system (Drury et al. 2017), and frequency of drive-resistant alleles (Champer et al. 2017, Drury et al. 2017). Moreover, CRISPR/Cas9 HDR is dependent on the choice of DNA double-strand break repair via homologous recombination versus non-homologous end joining (NHEJ). HDRis necessary to permit the conversion of a targeted locus and thus subsequently change inheritance frequencies. Whereas, if NHEJ occurs, it tends to be error prone, creating by insertion or deletion new alleles which are refractory to a gene drive system. Moreover, HDR is restricted to the S and G2 phases of the cell cycle, while NEJH is active throughout the cell cycle (Brandsma and Gent 2012). Therefore, CRISPR/Cas9 HDR based mechanisms are not only dependent on the molecular mechanisms of repair but also on unique aspects of the species of interest.

*Tribolium castaneum*, like *Drosophila*, is an established model organism for developmental, evolutionary, and applied (e.g., insecticide) biology but is also a known agriculture pest throughout the world (Beeman et al. 1989, Brown et al. 2009, Adamski et al. 2019, Rosner et al. 2020, Pointer et al. 2021, Campbell et al. 2022, Klingler and Bucher 2022). Hence, to demonstrate a proven methodology for CRISPR/Cas9 HDR, and subsequently, its application to gene drive would be beneficial to the research community. The genetic tools available to *Tribolium* include transgenesis, RNAi, and CRISPR/Cas9 genome editing (Klingler and Bucher 2022). Cas9 editing has been achieved by injection of ribonucleoprotein (RNP) complexes (Adrianos et al. 2018, Shirai and Daimon 2020, Shirai et al. 2022), plasmid encoded reagents (Gilles et al. 2015, 2019, Rylee et al. 2018), and injection of plasmid encoded gRNAs into transgenic *Tribolium* expressing Cas9 (Rylee et al. 2022). CRISPR/Cas9 edited genes include but are not limited to *vermilion* (Adrianos et al. 2018, Rylee et al. 2022), *E-cadherin* (Gilles et al. 2015), *cardinal* (Shirai and Daimon 2020, Shirai et al. 2022), and *foxQ2* (He et al. 2019) as well as the ability to target inserted exogenous sequences like GFP (Gilles et al. 2015, 2019). Whereas the capability to utilize CRISPR/Cas9 edit the genome is established in *Tribolium*, the extent and possibility of HDR is limited to 3 reports and with various success/efficiency (Gilles et al. 2015, Rylee et al. 2018, Farnworth et al. 2020). Here, we attempted to demonstrate the ability of CRISPR/Cas9 dependent homologous recombination and possibly gene drive in *Tribolium*. Our results indicate that even though genome editing was achieved, our injections did not result in any recovered HDR modifications, only NHEJ repair, and thus subsequent methodologies and modifications will be needed to detect and increase a bias towards HDR in *Tribolium*.

## Materials and Methods

### Tribolium Husbandry and Strains

All animals were raised at 28 °C on a standard flour yeast mix. The following strains were utilized: *vermilion*^*white*^ (*v*^*w*^), (Lorenzen et al. 2002), GA-1, and Henderson Black (HB) (Ruckman and Blackmon 2020).

### Vectors and gRNA Sequences

#### Tc-v 1gRNA Backbone Drive Homology Construct

The following components were synthesized and cloned into pUC57 (Synbio Technologies). *vermilion* left homology arm (1,000 bp), U6a promoter driving the expression of Tcv95 gRNA (Adrianos et al. 2018), AscI cloning site, and *vermilion* right homology arm (1,000 bp).

#### Tc-v 2gRNA Backbone Drive Homology Construct

The following components were synthesized and cloned into pUC57 (Synbio Technologies). *vermilion* left homology arm (1,000 bp), U6a promoter driving the expression of TcV95 gRNA (5ʹ-AAATTAAGTGAAGCCCAAGAAGG-3ʹ) (Adrianos et al. 2018), U6b promoter driving expression of TcV412 gRNA (5ʹ-GGATCAAAACAACACGATTGAGG-3ʹ), AscI cloning site, and *vermilion* right homology arm (989 bp).

*hsp68-nls-Cas9-hsp3ʹUTR* cassette was excised from p(bhsp68-Cas9) (Gilles et al. 2015) Addgene (#65959) using flanking Asc1 sites and ligated into the AscI of constructs containing either 1 or 2 gRNA homology repair drive constructs.

*nanos-nls-Cas-9-T2A-EGFP-nanos UTR* cassette was flanked by AscI sites synthesized and cloned into pUC57 (Synbio Technologies). The potential *nanos* promoter consists of 347 bp of the first coding Methionine of *nanos* and *nanos* 3ʹUTR sequence is represented by 406 bp of DNA immediately downstream of the *nanos* termination codon. The Cas9 cassette was excised using flanking Asc1 sites and ligated into the AscI of both homology repair drive constructs.

All sequences of plasmids injected can be found in Supplement Material as.gb files.

Tribolium CRISPR injections: Injections were performed at 25 °C and embryos were then returned to 28 °C for development and hatching. Each construct was resuspended in 1× injection buffer (0.5 mM KCl; 0.01 mM NaPO4 buffer pH 7.5) at a concentration of 1 µg/µL and injected into GA-1 or HB embryos. Individual injected G0 males were mated to 2–3 *v*^*w*^ females and individual injected G0 females were mated to 2–3 *v*^*w*^ males. Progeny were then subsequently screened for the loss of pigment in the eye. The progeny from each individual founder G0 cross (G0 × *v*^*w*^) that generated *vermilion*, non-pigmented eyes, were saved for future analyses. To test for gene drive, two individual *vermilion* progeny from each positive G0 founder were crossed to the pigmented HB or GA-1 strain. If gene drive was present, we would expect to see *vermilion*, non-pigmented individuals in the progeny from this cross. Second, additional individual F1 *vermilion* progeny from each positive G0 founder individual were tested for the presence of Cas9, utilizing PCR primers directed against a 579 bp of the Cas9 coding region. In addition, samples were subjected to PCR and sequenced for the identification and confirmation of CRISPR/Cas9 editing.

PCR and Sequence Confirmation of CRISPR/Cas9 editing: Genomic DNA was isolated from individual animals by crushing an individual in 50 µl of extraction buffer (100 mM Tris-HCl, 50 mM EDTA, 1% SDS) with a pestle in an Eppendorf tube. The mixture was subjected to a 5 min incubation at 95 °C, then chilled on ice. The mixture was then digested with Proteinase K (50 µg/mL) for 1 h at 55 °C, followed by heat inactivation at 95 °C for 5 min. 200 µl of 0.1× TE buffer was added to dilute the sample. Finally, 100 µl of the gDNA solution was purified using the Zymo Genomic DNA Clean and Concentrator-10 kit (Zymo Research #ZD4010) following the manufacturer’s instructions. Amplicons spanning the gRNA target sites were amplified from 1 µl of purified gDNA using HotStar PCR Master Mix (Qiagen). Half of each reaction was run on a 1.5% agarose gel, and the other half of the reaction was purified using the Qiaquick Gel Extraction Kit (Qiagen). The purified fragments were submitted to Eurofins Genomics for Sanger sequencing, and the sequences were analyzed using Sequencher (Gene Codes Corp.). The following primers were used to amplify the *vermilion* DNA flanking the targeted gRNA sites: 5ʹ-ACCTAAGGTCACGCGGAAGTATCGCATCGT-3ʹ and 5ʹ-CAGGAGCCTGAACTGCAGGCTCTGGAACCC-3ʹ and amplify an 806 bp fragment. The vermillion PCR products were sequenced with the following primer: 5ʹ-TATCGCTTTAGTTAGTCTAAA-3ʹ. The following primers were used to detect the presence of Cas9 5ʹ-CTCTAATCGAAACTAATGGGGAAACTGGAG-3ʹ and 5ʹ-GTTCGTTATCTTCTGGACTACCCTTCAACT-3ʹ and amplify a 579 bp fragment.

## Results

**Fig. 1.**
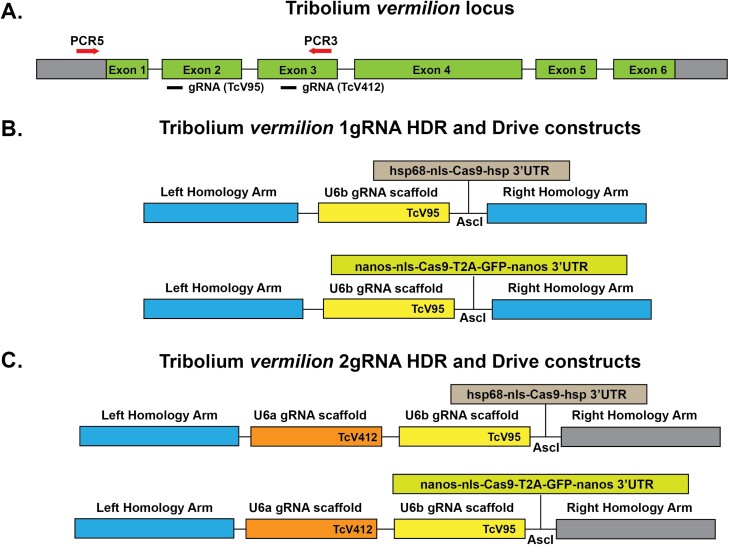
*vermilion* locus and Cas9 dependent homologous repair drive constructs. A) Schematic of the *vermilion* locus and location of gRNAs and PCR primers for amplifying the region surrounding the potential region of CRISPR/Cas9 editing. gRNA TcV95 and gRNA TcV412 are on different DNA strands. B) The two constructs for HDR and gene drive containing a single gRNA (TcV95). Cas9 was either expressed from the ubiquitous *heat shock protein 68 (hsp68)* promoter or the *nanos* promoter. The left homology arm (1,000 bp) extends from the upstream non-coding region of the *vermilion* locus to the gRNA and includes the gRNA sequence except for the PAM site. The right homology arm (1,000 bp) extends from the gRNA PAM site into exon 4. C) The two constructs for HDR and gene drive containing 2 gRNAs (TcV95 and TcV412). Cas9 was either expressed from the ubiquitous *heat shock protein 68 (hsp68)* promoter or *nanos* promoter. The left homology arm is identical to the left homology arm in the single gRNA construct B). The right homology arm (989 bp) includes the gRNA sequence except for the PAM sequence and extends into exon 5.

We chose to take a dual approach to examine for evidence of CRISPR/Cas9 homologous dependent repair and subsequent gene drive. Utilizing a process based on the mutagenic chain reaction (Gantz and Bier 2015), we developed a set of components to target the *vermilion* locus of *Tribolium*. We chose *vermilion* because it has been successfully targeted by CRISPR/Cas9 (Adrianos et al. 2018, Rylee et al. 2022), and loss of function mutations result in an easily scorable visible phenotype and loss of pigment in the adult eye. We generated 2 HDRback bone constructs in which various versions of Cas9 can be inserted. The backbone constructs differ in the number of gRNAs expressed, 1 versus 2, and subsequently, different sequences for the right homology arms based upon the position of the second gRNA utilized (Fig. 1). The HDR backbones were designed to disrupt the open reading frame of *vermilion*. The single gRNA backbone contained the TcV95 guide RNA (Adrianos et al. 2018) and TcV95 has previously been demonstrated to guide Cas9 for genome editing. The two guide RNA backbone contained TcV95 a second gRNA, TcV412, which had not been tested previously. For Cas9 expression we utilized 2 different Cas9 expression vectors. The first was the established hsp68Cas9 cassette (Gilles et al. 2015), where Cas9 expression is under the control of the core hsp68 gene promoter, presumably active in both the germline and soma of *Tribolium* and does not need a heat pulse for expression and contains a single nuclear localization signal on the 5ʹ end of Cas9 (Schinko et al. 2010). In addition, the Cas9 has the associated 3ʹ UTR of *hsp68*. The second Cas9 cassette has Cas9 expression controlled by the putative promoter for *Tribolium nanos*. In *Drosophila*, *nanos* is a germline specific transcript and thus, this cassette could potentially limit Cas9 expression to the germline. However, this exact role of *nanos* in *Tribolium* has not been demonstrated but *T. nanos* is required for posterior patterning (Schmitt-Engel et al. 2012). In addition, the construct has the associated 3ʹ UTR of *nanos*, which in *Drosophila* is necessary for both localization and translation at the embryonic posterior pole (Gavis and Lehmann 1992, 1994). Overall, four different constructs were injected. For injections, we chose to inject wild-type *Tribolium* strains that had pigmented eyes, HB, and GA-1. For the majority of injections, GA-1 was utilized due to its apparent but not quantified greater fecundity; embryos were readily available for injections from the GA-1 stock but not from HB.

For each construct, at least 2 rounds of injections were completed. Surviving injected individual males and females were outcrossed to *v*^*w*^ beetles. v^w^ contains a large deletion that removes most of the *vermilion* locus and results in non-pigmented eyes (Adrianos et al. 2018). As such, in the F1 generation potential HDR and drive candidates were identified by the loss of pigment in the eye. For all 4 constructs, non-pigmented F1 progeny were identified (Table 1). In any particular cross that generated non-pigmented progeny, the number of F1 *vermilion* progeny ranged from a few to over 50%.

**Table 1.** Summary of injections and recovered *vermilion* edited progeny

Construct injected	Genotype injected	# of G0 individuals		# of G0 individuals with *vermilion* progeny		% of G0 individuals with *vermilion* progeny
		Female	Male	Female	Male	
*hs-Cas9 1gRNA*	Henderson Black	32	33	4	13	26.1%
*nanos-Cas9 1gRNA*	GA-1	69	77	4	3	4.8%
*hs-Cas9 2gRNA*	GA-1	124	95	8	5	5.9%
*nanos-Cas9 2gRNA*	GA-1	56	70	1	6	5.5%

Upon the identification of *vermilion* progeny, 2 subsequent analyses were conducted. To test for the presence of homologous recombination and subsequent functional drive, 2, where possible a male and a virgin, *vermilion* F1 progeny were crossed to GA-1 pigmented individuals. These F1 would be heterozygous for any insertion of the HDR construct or for incorrect repair of the *vermilion* locus via NHEJ and the second allele would be *v*^*w*^. If HDR did occur and genetic drive was active, we would expect 50% of the resulting progeny to contain non-pigmented eyes, the result of inheriting the HDR allele, which then was expected to mutate the wild-type *vermilion* allele inherited from the GA-1 parent. The other 50% of progeny were expected to be pigmented as a result of inheriting maximally 1 *v* allele and the presence of a wild-type *vermilion* allele from the GA-1 parent. None of the presumed F1 CRISPR edited *vermilion* progeny, when crossed to GA-1 resulted in the presence of *vermilion* progeny, suggesting incorrect repair of the *vermilion* locus via NHEJ and a lack of drive. However, our constructs do lack a visible marker, e.g 3XP3-Fluoresence, and thus relied on drive as an indicator of HDR. These results combined suggested that CRISPR/Cas9 directed HDR did not occur.

**Fig. 2.**
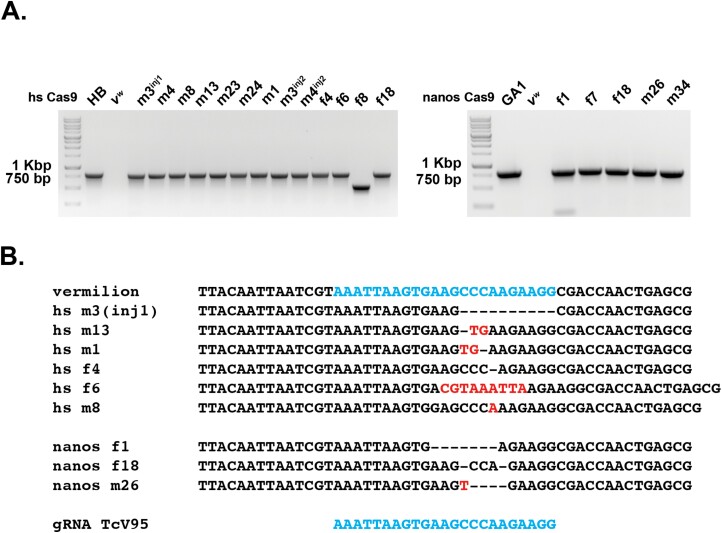
PCR and Sequencing results of CRISPR/Cas9 editing utilizing a single gRNA combined with Cas9 expressed from the *hsp68* or *nanos* promoters. A) PCR amplicons from isolated *vermilion* progeny from injections with the HDR constructs containing 1 gRNA and Cas9. The PCR amplicons are relatively equal in size or smaller to the expected to the unedited *vermilion* locus, demonstrating the lack of homologous recombination in the *vermilion* progeny. The numbers above each lane refer to the GO injected individual, m-male and f-female. B) Alignment, sequence confirmation, identificati,on and nature of CRISPR/Cas9 editing in a sample of the PCR amplicons from the isolated *vermilion* progeny. Dashes represent deleted bases and red color bases indicate insertions.

**Fig. 3.**
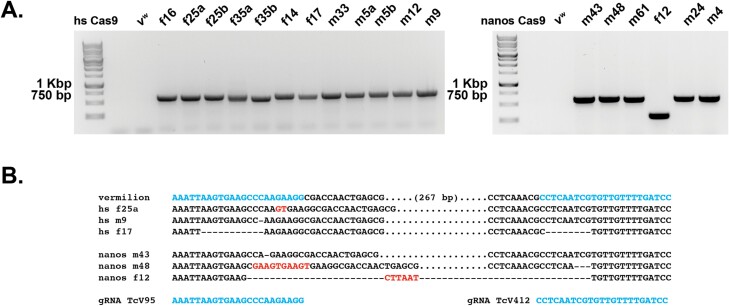
PCR and Sequencing results of CRISPR/Cas9 editing utilizing a 2 gRNAs combined with Cas9 expressed from the *hsp68* or *nanos* promoters. A) PCR amplicons from isolated *vermilion* progeny from injections with the HDR constructs containing 2 gRNAs and Cas9. The PCR amplicons are relatively equal in size or smaller to the expected unedited *vermilion* locus, demonstrating the lack of homologous recombination in the *vermilion* progeny. The numbers above each lane refer to the GO injected individual, m-male and f-female. B) Alignment and sequence confirmation and identification and nature of CRISPR/Cas9 editing in a sample of the PCR amplicons from the isolated *vermilion* progeny. f12, represents a deletion that spans between the 2 gRNA target sites. Dashes represent deleted bases. Red color bases indicate insertions and a dot represents base pairs that are present but not shown.

Lastly, we needed to confirm that the *vermilion* progeny obtained were the result of CRISPR/Cas9 editing and with respect to the 2 gRNA constructs, do both gRNAs work and can we detect editing with both gRNAs on the same DNA molecule? For each F1 progeny from the G0 injected individuals that resulted in *vermilion*, non-pigmented eyes, genomic DNA was isolated, and primers were used to amplify DNA spanning across the location of the directed gRNA cuts (Fig. 1). For those samples that resulted in a PCR product (Figs. 2A and 3A), the PCR product was sequenced and examined for changes in the DNA. Some samples did not result in a PCR product, possibly suggesting the CRISPR modification affected 1 or both PCR primer sites. Alternatively, NHEJ or incomplete HDR could have resulted in insertion of parts of the HDR construct, or HDR did occur, but drive was not functional. All 3 possibilities would hamper the appearance of a PCR product. To test these possibilities, we examined each F1 individual that lacked a PCR amplicon for the presence of Cas9 via PCR of a small, 579 bp, amplicon of the coding region of Cas9. We did not detect the presence of Cas9 in any of these samples (data not shown).

With respect to the samples that did result in a PCR product, as expected, the 1 gRNA backbone HDR construct with either Cas9 under the control of *hsp68* or *nanos* resulted in deletions, insertions or a combination of both (Fig. 2B). With respect to the 2-gRNA backbone HDR constructs, we found examples in which a single gRNA was utilized and examples of DNA molecules that contained evidence of CRISPR/Cas9 editing at both targeted sites on the same DNA molecule with either *hsp68* or *nanos* promoter driving Cas9 expression (Fig. 3B). Furthermore, we recovered 1 edited mutation, f12, that deleted the entire region, except for the insertion of 6 nucleotides, between the 2 gRNA target sites (Fig. 3B). Overall, our results demonstrated that both HDR backbone constructs, gRNAs, and Cas9 expression cassettes can induce double strand breaks, the individual gRNA RNP complexes can induce changes independently of each other and the lack of HDR may not be due to the functionality of the designed components.

## Discussion

There are many possibilities for the failure to detect HDR and subsequent gene drive. However, with respect to our study, a couple stand out. First, our detection of HDR was dependent on a second functional event, gene drive versus the detection of a visible marker (Gilles et al. 2015). To date, there have not been any reports of successful CRISPR/Cas9 induced gene drive in *Tribolium*. Moreover, our small sample size for detection of HDR could have hampered our detection of HDR given the reported low frequencies of HDR (Gilles et al. 2015, Rylee et al. 2018, Farnworth et al. 2020). Furthermore, we did not test every individual F1 *vermilion*, non-pigmented progeny, from each individual G0. Any injected G0 can give rise to several different CRISPR/Cas9 gene edits and thus only sampling some of the F1s from each G0 further reduced our sample size and the probability of detecting a HDR event.

Nonetheless, our study has demonstrated and reinforced a few key principles that will aid future editing in *Tribolium*. We have demonstrated the functionality of our gRNAs and the ability to direct Cas9 editing to 2 sites on the same DNA molecule. If we compare the use of one versus 2 gRNAs with respect to generating a mutant phenotype, non-pigmented eyes, we observe only a small difference in our results, 4.79% (nanos–1 gRNA) versus 5.56% (nanos–2 gRNA). But the simultaneous utilization of 2 gRNAs did generate a deletion between the 2 gRNAs and thus raises the possibility to make specific deletions in the genome. Interestingly, we did observe a greater CRISPR/Cas9 efficiency in the HB strain, but we cannot speculate why, and the result would need to be repeated. Besides, as previously mentioned, the fecundity, and the ability to get a reasonable number of embryos to inject was difficult from the HB strain.

Furthermore, we have shown the functionality of our *nanos* Cas9 cassette, providing a second methodology to express Cas9. However, 1 key caveat is that we do not have any evidence that our *nanos* Cas9 construct is specifically expressed in the germline. The activity of Cas9 observed may simply be due to spurious transcription from a core promoter contained within the genomic DNA utilized. The efficiency of generating CRISPR/Cas9 edited alleles of *vermilion* in our experiments is 8% of surviving G0 progeny. It is difficult to directly compare with other studies given the differences in methodology and the variability of the injections themselves. Even though an unbiased study would be required to confirm, we feel the inclusion of both the gRNAs and the expression cassette for Cas9 on the same plasmid as a general approach guarantees that all the components required for CRISPR/Cas9 are expressed together in any particular cell and thus possibly increasing the efficiency.

Is there a possible methodology to skew repair towards HDR versus NHEJ in future work? One possibility would be to eliminate the function of Lig4; Lig4 is a critical enzyme for NHEJ (Teo and Jackson 1997, Wilson et al. 1997). In *Drosophila*, *Lig4* mutants are homozygous viable (McVey et al. 2004) and editing of the genome with zinc-finger nucleases or CRISPR/Cas9 viable HDR was biased towards HDR (Beumer et al. 2008, Gratz et al. 2014). In addition, RNAi depletion of Lig4 in *Drosophila* tissue culture cells increased the frequency of HDR versus NHEJ when utilizing CRISPR/Cas9 (Bottcher et al. 2014). In *Tribolium*, RNAi knockdown of Lig4 did not significantly improve knock-in HDR, but the authors could not eliminate the possibility that maternal deposited Lig4 protein was accounting for this result (Gilles et al. 2015). Interestingly, *Tribolium* has not 1 but 2 orthologs of *Lig4* (LOC657210 and LOC657043) and thus future experiments may require mutating one or both and testing whether NHEJ with respect to CRISPR/Cas9 editing is decreased with subsequent increase in the frequency of HDR. Moreover, in *Drosophila* HDR frequencies were the greatest when plasmids encoding the gRNAs and the homology repair template were injected together into *Drosophila* transgenic lines expressing Cas9 in the germline (Gratz et al. 2014). Currently in *Tribolium*, there is not a well-established transgenic Cas9 with expression limited to the germline, but a transgenic version of the hs-Cas9 cassette exists (Rylee et al. 2022) and can be used in future CRISPR/Cas9 HDR attempts and may increase HDR efficiencies.

## Data Availability

All relevant data are within the paper and its Supporting Information files. All vectors generated are available through the Drosophila Genomics Resource Center at Indiana University and complete sequences of injected constructs can be found in Supplement Files.
